# An Action Field Theory of Peripersonal Space

**DOI:** 10.1016/j.tics.2018.09.004

**Published:** 2018-12

**Authors:** Rory J. Bufacchi, Gian Domenico Iannetti

**Affiliations:** 1 Department of Neuroscience, Physiology and Pharmacology, University College London, London, UK; 2 Centre for Mathematics and Physics in the Life Sciences and Experimental Biology (CoMPLEX), University College London, London, UK; 3 Neuroscience and Behaviour Laboratory, Istituto Italiano di Tecnologia, Rome, Italy

**Keywords:** perception and action, action selection, egocentric coding, motor system, defence, goal-oriented actions

## Abstract

Predominant conceptual frameworks often describe peripersonal space (PPS) as a single, distance-based, in-or-out zone within which stimuli elicit enhanced neural and behavioural responses. Here we argue that this intuitive framework is contradicted by neurophysiological and behavioural data. First, PPS-related measures are not binary, but graded with proximity. Second, they are strongly influenced by factors other than proximity, such as walking, tool use, stimulus valence, and social cues. Third, many different PPS-related responses exist, and each can be used to describe a different space. Here, we reconceptualise PPS as a set of graded fields describing behavioural relevance of actions aiming to create or avoid contact between objects and the body. This reconceptualisation incorporates PPS into mainstream theories of action selection and behaviour.

## Highlights

Many behavioural and neurophysiological responses depend on the proximity of environmental stimuli to the body or to specific body parts.

This has led to the intuitive, but ill-defined, concept of PPS as a single, distance-based, in-or-out zone. However, this intuitive framework is contradicted by empirical data.

PPS is instead better conceptualised as a set of several different graded fields, affected by many factors other than stimulus proximity.

PPS fields emphasize different behavioural functions and, thus, are better understood as mappings onto behaviour, rather than as representations of stimulus configuration.

This reconceptualisation incorporates PPS into mainstream theories of action selection and behaviour.

## What Is Peripersonal Space?

Interactions occurring within the space near the body have been studied in a range of disciplines, including ethology, neurophysiology, social science, architecture, and philosophy 1, 2, 3, 4, 5. Such studies have shown that many behavioural responses are increased when stimuli occur near the body. This phenomenon makes evolutionary sense: a predator within striking distance is more relevant than one farther away. Neuroscientific studies in both primates and humans have suggested a physiological foundation for such behavioural modulations, leading to the concept of peripersonal space (PPS).

But what is precisely meant when referring to PPS? This seemingly naïve question is in fact not easy to answer, as demonstrated by the great deal of terminological and conceptual confusion in the field (Box 1). A clear conceptual framework is lacking. A current and predominant perspective on PPS describes it as a single, distance-based, in-or-out space. While a sharp spatial boundary may be intuitively appealing, neurophysiological and behavioural data contradict the description of an in-or-out space. For example, many PPS-related neurons respond to stimuli with graded or even reverse relationships to distance 6, 7. Behavioural responses in humans are also graded with distance 8, 9, 10. These findings challenge a simple in-or-out definition. There is also reason to question a purely distance-based definition of PPS. PPS responses are influenced by factors such as walking, motion of body parts, tool use, stimulus trajectory, and stimulus valence 11, 12, 13, 14, 15. Finally, PPS is often presented as ‘the’ PPS, implying it to be a single entity. However, many different PPS-related responses exist 8, 16, and each can be used to describe a different space. These facts call the notion of a single PPS into question.

Box 1 **Definitional Issues with Peripersonal Space**

Here, we provide examples of the terminological and conceptual confusion in the existing literature about peripersonal space (PPS). This term is typically used with three different meanings, as described below.

Meaning 1: the portion of space within a given Euclidian distance of the body (e.g.: ‘[…] the space immediately around the animal (peripersonal space)’ [22]).

Meaning 2: the space within which certain physiological or behavioural responses are larger when the stimuli eliciting them occur near the body (e.g. ‘The spatial extent to which visuotactile interactions strongly occur is known as visuotactile peripersonal space (hereafter called “peripersonal space”)’ [83]).

Meaning 3: the mechanisms through which the brain represents the space near the body (e.g. ‘a multisensory representation of the space surrounding the body, i.e. the peripersonal space (PPS)’ [85]).

While meaning #1 poses no problems, it is not physiologically interesting: in this meaning, PPS is immutable and exists regardless of the individual being alive or dead, and therefore is not adequate to explain the phenomenon that PPS can change in size. When this occurs, meanings #2 and/or #3 are invoked. However, a serious problem arises when meanings #2 and #3 are considered equivalent. Typically, when it is observed that a physiological response function changes with proximity (i.e., PPS in meaning #2), it is also presented as evidence that the neural representation of near space (i.e., PPS in meaning #3) expands, contracts, or changes shape. However, this equivalence between ‘the space within which certain physiological or perceptual responses are larger when the stimuli eliciting them occur near the body’ and ‘the mechanisms through which the brain represents the space near the body’ is false. While it is true that a measure dependent on spatial proximity (meaning #2) tells us something about how the brain represents near space (meaning #3), it is not true that a modulation of the measure dependent on spatial proximity (meaning #2) necessarily means that the brain has changed how it represents near space (and that ‘PPS has expanded/contracted’; meaning #3). In fact, this false equivalence can even be found when PPS is initially defined geometrically (i.e., using meaning #1), but is later said to change in size or shape to accommodate observations. These various definitions are often used even within the same sentence, for example ‘Peripersonal space (PPS), defined as the space immediately surrounding the body [meaning #1], is now well accepted as a region of integration of somatosensory, visual, and auditory information [meaning #2]. It is a privileged interface for interaction with nearby objects [meaning #3]’ [86]. Notably, we are not exempt from this criticism ourselves: ‘The defensive peripersonal space (DPPS) is a portion of space surrounding the body [meaning #1] with a crucial protective function [meaning #3]. Whenever a potentially dangerous stimulus approaches or enters this area, the individual engages in protective actions aimed at avoiding or minimising harm [meaning #2]’ [42]. Such conflations of meanings are likely to cause conceptual misunderstandings.

Alt-text: Box 1

While conceptualising PPS as a single distance-based in-or-out zone may allow for efficient summary of results, it is increasingly clear that this simple framework has become a source of data misinterpretations and conceptual misunderstandings. Here, we offer a new framework. Rather than describe PPS simply as an in-or-out zone, we propose reconceptualising PPS as a set of graded response fields. Each field describes the magnitude of a certain physiological or perceptual measure that reflects the behavioural relevance of a stimulus to a given action or set of actions. Specifically, we refer to those actions that aim to either create or avoid contact between objects and the body.

This framework contains three concepts with important implications: (i) a field allows PPS measures to change gradually with distance, rather than to define an in-or-out space; (ii) a set of fields reflects the fact that there are many different PPS measures showing different response profiles; and (iii) behavioural relevance to actions aiming to create or avoid contact between objects and the body explains the functional significance of the values composing the PPS field of each action, and the fact that factors other than proximity affect PPS measures. We believe that this framework can explain seemingly anomalous empirical observations, and resolve some of the definitional and conceptual issues affecting the field.

## More Than an ‘In-or-Out’ Bubble

In probably the first observations of a link between brain function and the processing of sensory stimuli occurring near the body, the British neurologist Lord Brain [17] noticed that certain lesions of parietal cortex led to perceptual problems in near space, but not in far space. During the 1950s, the Swiss zoologist Heini Hediger observed that behavioural responses depended upon the distance of the triggering stimulus. He reported that stimuli near the animal (inside a so-called ‘flight zone’) elicited a flight response, whereas stimuli outside the flight zone elicited no response 4, 18. In 1969, the influential anthropologist Edward Hall, the father of proxemics, published a book about the importance of space to human behaviour [3]. He distinguished between intimate space (up to 45 cm), personal space (up to 1.2 m), social space (up to 3.6 m), and public space. While their empirical evidence did not support the existence of multiple distinct spaces, these authors nonetheless opted to present their findings as though there were multiple distinct spaces, probably to ease the understanding of the reader. While informative, these early seminal studies therefore implicitly set the tone that near and far space have sharp boundaries. Studies and interpretations over the next several decades adopted this simple framework. However, a closer look at many of these studies challenges such a framework.

**Figure 1:**
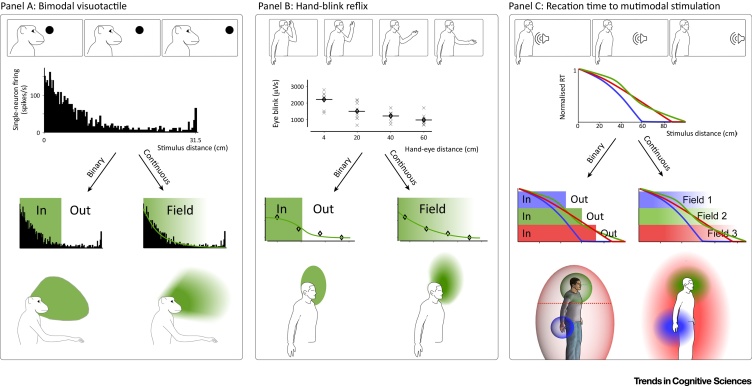
Describing Peripersonal Space (PPS): Gradient or Boundary? Many behavioural and neurophysiological responses have been labelled as PPS measures because their magnitude increases with body proximity. However, even when clearly graded with proximity to a body part, PPS measures are often described using binary ‘in-or-out’ metrics and wording (left side of each panel). This approach often consists in choosing some cut-off value to define the PPS ‘size’. Examples of such cut-off values are the furthest distance at which consistent modulation is observed (A) and the midpoint of a fitted function (B,C). (A–C) show examples of how PPS data could be described as binary ‘in-or-out’ metrics [left side of (A–C)], but more faithfully reflect the data when displayed as a continuous, graded response field [right side of (A–C)]. (A) Bimodal visuotactile neurons fire more when visual stimuli are close to their tactile receptive fields [25]. However, as discussed in the main text, this is an oversimplified description. (B) The hand-blink reflex is elicited by stimulation of the median nerve at the wrist, and increases in magnitude when the hand is closer to the face [35]. (C) Reaction times (RT) to somatosensory stimuli on the face (green), hand (blue), and chest (red) are faster when auditory or visual stimuli are concomitantly presented closer to those body parts [38]. Other types of visuotactile and audiotactile integration have also been shown to increase in magnitude with proximity the body [87]. Data reproduced, with permission, from 9, 25, 88.

For example, studies of bimodal single neurons in macaques illustrate how data and interpretation often disconnect 7, 19, 20, 21, 22, 23. In these studies, bimodal neurons were identified in both cortical and subcortical structures (putamen, parietal, and premotor areas). These neurons responded not only to somatosensory stimuli, but also to visual or auditory stimuli presented in spatial proximity to the somatosensory receptive field. The authors emphasised the larger neural response magnitudes to stimuli in near space versus the smaller neural response magnitudes to stimuli in far space. To illustrate these findings, such neurons were often described as demarcating zones of space, represented as bubbles with clear boundaries [24] (Figure 1). However, these boundaries were simply lines of arbitrary response magnitude [25]. Other authors similarly defined such boundaries as the ‘part of space which gave consistent responses’, again resulting in arbitrary and ill-defined in-or-out zones [26]. The fact that some neurons did respond preferentially to stimuli occurring near a given body part was presented as the most novel and memorable aspect of these studies. Therefore, on the surface, these neurophysiological studies appeared to confirm, at the neural level, the conception by seminal studies of a sharply defined ‘in-or-out’ near space.

However, the data in these same studies clearly illustrate that response magnitudes are not simple step-like functions but have far more complex properties. In many cases, the responses appear to be gradual, rather than step-wise, proximity functions 6, 7, 21, 26, 27, 28 (Figure 1). Indeed, while the response magnitude of some neurons increases rapidly with stimulus proximity between two consecutive tested points, thus defining what appears to be a ‘sharply delimited receptive field’ [7], most neurons show a less steep response gradient [7]. In addition to this graded response, a sizeable portion of multimodal neurons (e.g., ∼5% of recorded neurons in ventral premotor cortex [24]) exhibit receptive fields that extend further than reaching distance, sometimes even to the end of the room 7, 25. Therefore, neuronal response functions are not only more continuous than usually presented, but some of these response functions also encompass a much larger area than commonly reported. Finally, some neurons even show the reverse of the expected near versus far relationship: in both premotor and parietal regions, they respond less strongly to visual stimuli near their somatosensory receptive field than to visual stimuli slightly further away in space 7, 21, 26. Taken together, these empirical data demonstrate that a step-like proximity function is an inadequate description of PPS.

These interpretive issues are not limited to neurophysiology experiments: the psychophysical literature suffers analogous oversimplifications. At first, most psychophysical experiments would often test only two conditions: near versus far. Such ‘near versus far’ experiments were performed using various behavioural measures, such as line bisection [29], visuotactile extinction [30], audiotactile extinction [31], visuotacile interaction [32], the hand-blink reflex (HBR) [33], and temporal-order judgements of nociceptive stimulation [34]. The desire to keep experimental design and analysis simple is understandable, and not necessarily a fatal flaw. Indeed, most researchers are aware that the responses change continuously between the sampled near and far positions: sampling two points along a continuum does not negate that continuum. However, the binary experimental design implies a binary response pattern, precluding a more complete conceptual understanding of PPS.

Even in the subset of psychophysical experiments where more stimulus locations were tested, and the corresponding responses clearly showed a graded fall-off, authors often continued to present results as ‘in-or-out’ zones using simple summary statistics, such as the point of first increase over baseline activity [35], or the mid-point of a fitted sinusoidal function 36, 37, 38. Even when it is explicitly made clear that changes in response magnitude from near space to far space are gradual, for example in line-bisection work 39, 40 and work on the HBR 9, 35, 41, 42, the discussion is still often framed as ‘near space versus far space’.

## More Than Proximity: Many Variables Influence PPS Measures

Although proximity to the body or a body part is a crucial factor determining the spatial properties of PPS measures, it has become clear through recent experimental work that many other factors affect these measures.

Indeed, in addition to proximity, PPS responses are affected by stimulus movement parameters, such as speed and direction. This phenomenon had already been observed in the original recordings of bimodal neurons in macaques. For example, ∼80% of neurons in the ventral-intraparietal area (VIP) respond more than twice as much to stimuli moving in a preferred direction [7]. Even bimodal visuotactile neurons selectively responding to looming stimuli do not necessarily have monotonic response functions. While the majority of these neurons do respond most strongly to stimuli in the nearest stimulus position tested (e.g., 5 cm from the body part with the relevant tactile receptive field), some respond better to stimuli located at further distances (e.g., 20 cm from the somatosensory receptive field), and a few even further than that [7]. Furthermore, some bimodal VIP neurons respond preferentially to receding stimuli [6]. Interestingly, in these cells, the tactile response was triggered when the tactile stimulus was removed from, rather than applied to, the receptive field; what could colloquially be called ‘not-there-anymore’ neurons. In fact, VIP neurons include so much directional information that the borders of that area were originally functionally identified as the points at which direction-selective visual responses could be found 6, 7, 25. Recordings from VIP neurons can even be used to reliably decode self-motion and heading direction relative to the environment 43, 44, although, crucially, these neurons are not causally involved in heading perception [45]. Taken together, these observations show that the magnitude of PPS measures is not just determined by proximity. In fact, because of differential sensitivity to movement, two VIP neurons with the same tactile receptive field could show different response magnitudes even when the responses are elicited by identical visual stimuli. Cortical regions other than VIP also contain neurons sensitive to large numbers of stimulus features in addition to proximity, such as size, direction, speed, rotation, active and passive joint movement, and even the semantic value of the stimulus (e.g., some respond more strongly to snakes than to apples) 21, 25, 28, 46.

Recent findings in humans have also shown that many other factors not related to the current stimulus position can influence PPS-related neurophysiological and behavioural responses. These factors include walking 11, 47, gravitational cues [41], vestibular cues [48], motion of body parts 12, 49, 50, 51, stimulus direction [38], stimulus trajectory [15], and even higher-level factors, such as stimulus valence and semantics 14, 37, 52, 53 and environmental landscape [54]. PPS measures can also be affected by training or learning [16]. These effects, such as the well-characterised modulation of the response fields of multimodal neurons after tool use [13], provide additional strong support to the idea that stimulus proximity alone cannot adequately explain changes in PPS measures.

This wealth of additional factors determining PPS response magnitudes shows clearly that the concept of an in-or-out, near-or-far space leads to problems in reasoning. For example, when PPS measures are altered by stimulus movement, researchers have claimed that PPS or ‘the near space’ has changed in size. This has even been rephrased as ‘far becomes near’ [55]. However, given that many factors in addition to stimulus proximity can alter the PPS-related measure, the reverse inference underlying these claims is likely incorrect [56]. The reasoning has a clear element of circularity: ‘when a stimulus is close, the magnitude of a measure increases. Therefore, when the magnitude of a measure is increased, a stimulus is close’. This is not logically sound, because there can be factors other than proximity that cause the observation that the magnitude of the measure was increased. This insistence on proximity as the most important factor may turn out to be a historical legacy whose time has come. In other words, we have staked a larger importance on the factor proximity than on the factor movement direction, only because we have assumed that the measure denotes a space. In reality, there is no reason to think that, from a functional perspective, stimulus proximity is more important to PPS measures than any of the other factors they are sensitive to.

Interestingly, the same issue of the unjustified primacy of space might apply to the functional interpretation of place and grid cells. Classically, these cells have been considered to code the position of an animal relative to the environment, due to the strong correlation between their firing and the location of the animal 57, 58. However, recent evidence that these cells are not solely sensitive to spatial information [59] has led to the novel idea that place and grid cells do not represent space specifically, but instead code the value of a given state [60]. This is particularly relevant for the current discourse on PPS, not only because it provides evidence that a greater understanding of the system might be gained by revoking the primacy of spatial location, but also because the hippocampus and parieto-premotor loop likely work tightly together to plan and coordinate actions [61].

## More Than One Space: Many Different PPS Measures

**Figure 2:**
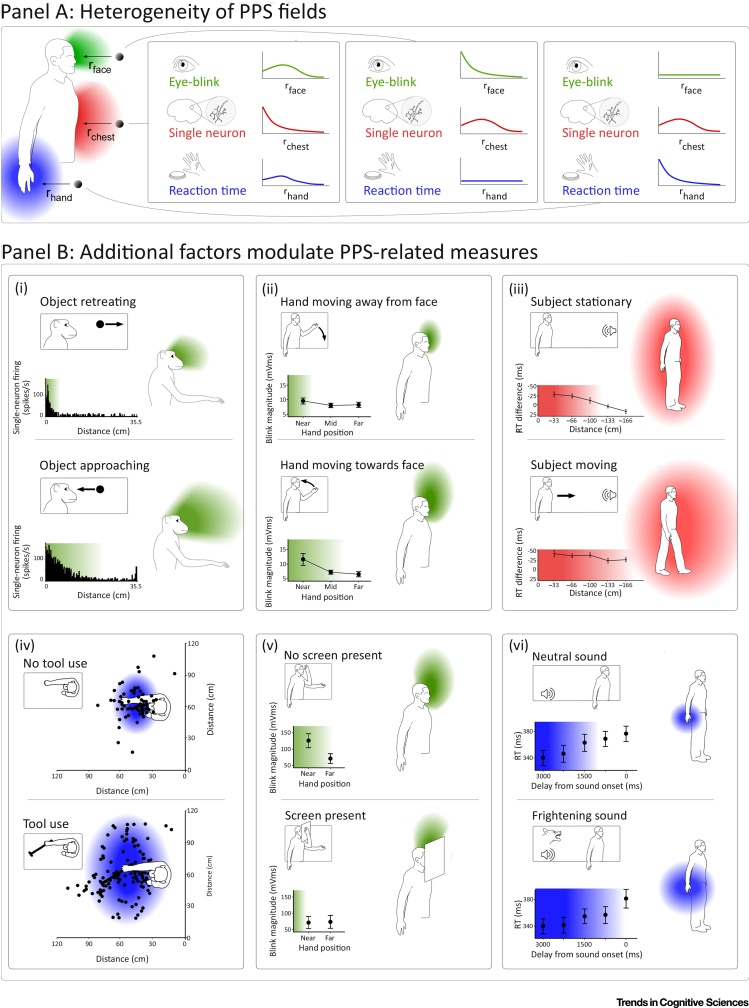
Many Peripersonal Space (PPS) Fields, Affected by Many Factors. As detailed in the main text, we propose a reconceptualisation of PPS as a set of fields reflecting the relevance of actions aimed at creating or avoiding contact between objects and the body. This figure illustrates the idea that there is not a single PPS, but that instead there are many PPS fields. (A) Heterogeneity of PPS fields. Different PPS fields can be derived from the many types of biological measure that differently depend on spatial proximity. Here, we show as an example the PPS fields derived from the modulation exerted by the proximity between a visual stimulus and the body on different biological measures: (i) the somatosensory-evoked eye blinking (green); (ii) the response of a visuotactile single neuron with a somatosensory receptive field on the chest (red); and (iii) the reaction times (RT) to somatosensory stimuli delivered to the hand (blue). Note how the same visual stimulus in an identical position elicits different responses and defines PPS fields with different spatial features. (B) Not just proximity: additional factors modulate PPS-related measures. Although the magnitude of PPS-related measures is commonly affected by proximity to a body part, many other factors also affect these PPS measures. Such factors include various types of motion: motion of a visual stimulus (i), stimulated limb (ii), and the entire body (iii) can all cause expansion of the response fields. Factors independent of motion also affect PPS measures: tool use can expand response fields (iv), a protective screen can deform them (v), and frightening sounds can expand them (vi). Response fields are colour-coded by the body part near which their magnitude is maximal: face (green), hand (blue), and trunk (red). Data reproduced, with permission, from 11, 13, 14, 25, 38, 54, 89.

As described above, many PPS-related measures exist. They range from audiotactile integration, which is maximal nearest the stimulated body part 38, 62, to the firing of different visuotactile macaque neurons, which increases when visual stimuli are closer to different body regions [19]. A feature that these measures have in common, and that has caused them to be grouped under the banner of PPS, is that their magnitude increases with proximity to a certain body part. However, what this body part is and how the magnitude depends on proximity changes from measure to measure. This is represented in Figure 2A, which shows how different types of biological measure, differently dependent on body proximity, yield different PPS fields. Furthermore, as described in the previous section, PPS measures are affected not only by proximity, but also by other factors, whose contributions to each measure vary.

Thus, due to the multitude of PPS-related response functions and their different sensitivity to factors different from body proximity, referring to ‘the’ PPS as if it were a single entity does not bring clarity to the discourse.

An attempt to reconcile the notion of a single PPS and the variety of PPSs derived from the many PPS-related measures is the suggestion that the single PPS is organised in a modular fashion [62]. However, this suggestion begs several difficult questions: in which manner does ‘the’ PPS arise from its various modules? Is it a weighted sum of all the PPS-related measures? And, if so, how are we to choose the weights? As detailed in the following section, we propose to shift perspective, consider the functional significance of the many PPS-related measures, and abandon the concept of a single PPS, for which there is currently a lack of empirical evidence.

## PPS as a Set of Contact-Related Action Fields

Here, we offer a reconceptualization of PPS that solves the issues arising from the notion that PPS is (i) a single entity; (ii) with a binary in-or-out boundary; and (iii) mostly dependent on stimulus proximity to the body. We propose that PPS be considered a set of continuous fields describing physiological or perceptual responses that reflect the behavioural relevance of actions aiming to create or avoid contact between objects and the body.

This formulation has several important concepts: field, a set (of fields), and contact-related behavioural relevance.

**Figure 3:**
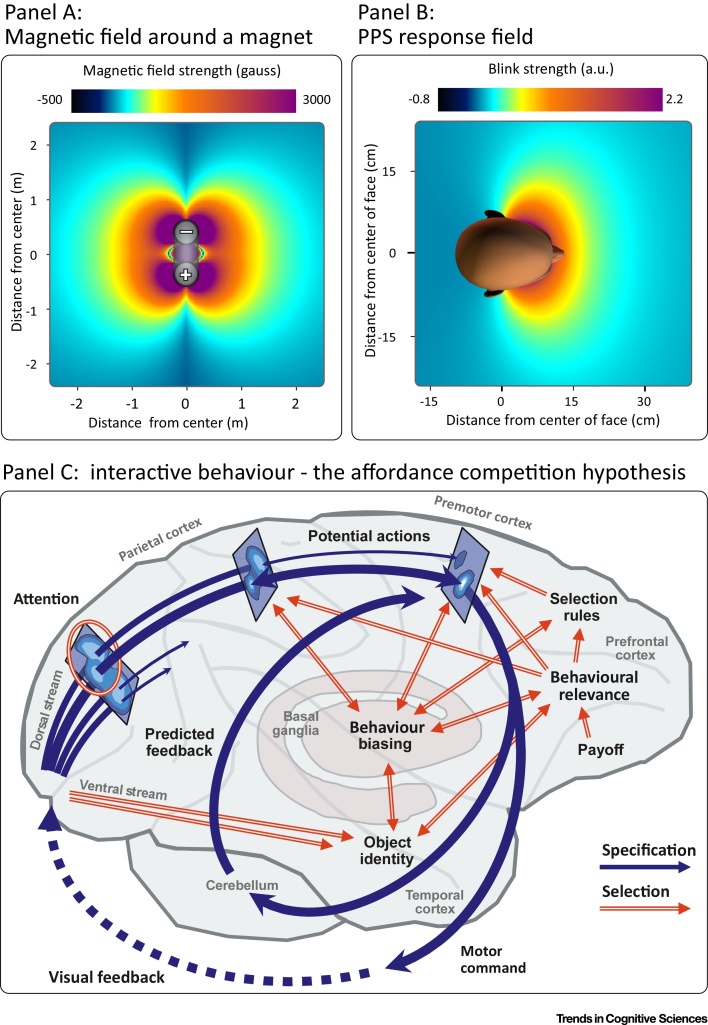
Peripersonal Space (PPS) as Action-Relevance Fields. We conceptualise PPS as a set of fields reflecting the relevance of actions aimed at creating or avoiding contact between objects and the body. We borrow the term ‘field’ from modern physics, to express a quantity that has a magnitude for each point in space and time [63]. (A) Magnetic field strength decreases proportionally to the cube of the distance from a coil. Therefore, when approaching a magnet holding a metallic object, one perceives a ‘boundary’ where the attractive force becomes detectable. Still, the magnetic field covers the entirety of space, although its strength becomes infinitesimal. (B) Similarly, PPS field values can be infinitesimal in most of the space far from the body, although this does not mean that they have zero value. Admittedly, considering such distant PPS field values is rarely phenomenologically useful, but it does become important in those instances of PPS measures with either extremely large response fields [24] or spatially separate regions with strong responses [90]. This panel shows the PPS field for the defensive eye blink [9]. Envisioning PPS as a set of response fields avoids the oversimplification of considering it a single and binary ‘in-or-out’ bubble, and it allows a richer description of the actual response properties in space. (C) The notion of contact-related action fields fits well with the interactive behaviour framework [71]. This framework, exemplified here for visually guided movements, postulates that neural populations in the parieto-premotor loop transform visual input into representations of potential actions. The strength of each action representation is also influenced by basal ganglia and prefrontal regions so that it reflects the relevance of that action depending on the environmental circumstances. Reproduced, with permission, from [71] (C).

We use the term ‘field’ in the same sense that it is used in modern Physics (Figure 3), which is to express a quantity that has a magnitude for each point in space and time [63]. Given that this magnitude can change continuously in space, the concept of a field accommodates the observations that PPS measures change gradually with distance in three dimensions. Importantly, a field does not necessitate PPS to be an ‘in-or-out’ zone, and allows multiple separated regions of nonzero magnitude.

The concept that there is not a single PPS field, but instead a set of fields, is compatible with the observation that different PPS measures show different response profiles. Rather than forcing us to choose which (or which combination) of the many PPS measures best represent ‘the’ PPS, or ‘what is near’, the concept of a set of fields allows us to see the various PPS measures as separate instances of a wider class of responses with some common functional significance.

The concept of contact-related behavioural relevance (i.e., the relevance of actions aiming to create or avoid contact between objects and the body) explains the functional significance of the values comprising a PPS field. Although the relevance of PPS to action has been stated and accepted by many, exactly how PPS is related to action remains unclear 11, 16, 24, 49, 50, 64, 65, 66, 67, 68, 69, 70. We propose that the values of PPS fields reflect the relevance of potential actions that aim to either create or avoid contact between a stimulus and a body part.

**Figure 4:**
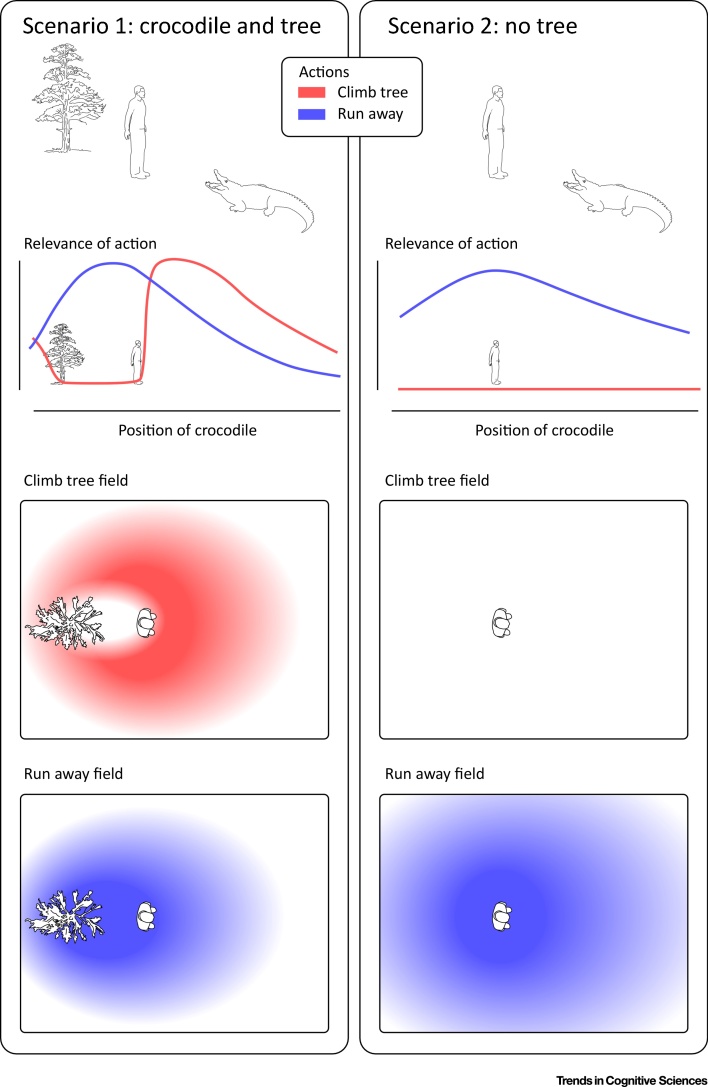
Effect of Context on Peripersonal Space (PPS) Fields. Consider two possible actions when facing a predator such as a crocodile: climbing a tree (red) or running away (blue). Given that the relevance of these actions depends on the position of the predator (second row), any measure reflecting the relevance of these actions can be used to map out a response field in space (third and fourth rows). Examples of these measures might be the probability of observing the given action, the firing rate of a neuron that is involved in preparing the action, or the reaction time to a sudden tactile stimulus on the body. In this perspective, the fields described by the magnitude of these measures are instances of PPS fields: fields that reflect the relevance of actions aimed at avoiding or creating contact. This conceptualisation (i) allows PPS measures to change gradually with distance; (ii) reflects the fact that many different PPS measures show different response profiles; and (iii) explains the functional significance of the values comprising the PPS field of each action, and the fact that factors other than proximity affect PPS measures. This example encapsulates those three points. In the presence of a crocodile and a tree (left column), climbing is an action the relevance of which increases with the proximity between the crocodile and the individual, unless the crocodile is interposed between the two (red). In the same situation, running away (blue) is a less beneficial action unless the crocodile is interposed between the tree and the individual. By contrast, in the absence of a tree (right column), climbing becomes irrelevant, while running away becomes more relevant.

The concept of contact-related behavioural relevance fits well with the perspective of interactive behaviour: rather than conceptualising behaviour as a stepwise process from sensory input to cognition to motor output, the interactive behaviour framework describes behaviour as a set of simultaneous processes specifying potential motor actions and selecting among them [71]. Notably, the parietal and premotor cortices (i.e., the cortices also thought to underlie the modulation of many PPS measures) are likely cortical sites where potential actions are specified within the interactive behaviour framework 61, 71, 72 (Figure 4). Furthermore, contact-related behavioural relevance also explains the relationship between many PPS measures and impact prediction 15, 73, 74: the probability of impact of a stimulus is strongly related to the behavioural relevance of an action aiming to avoid or create contact [9]. Interestingly, recent views have suggested that impact prediction is the main role of PPS [75]. While such a role does explain that some factors other than proximity (e.g., velocity or direction of movement) affect PPS measures, it still does not explain, for example, the effect of other factors, such as stimulus valence [14], lateral motion [7] (i.e., in a direction not likely to directly cause impact with the individual), and social interactions [76], factors that are instead explained by considering contact-related action relevance.

The large body of literature investigating PPS through behavioural and physiological measures provides many examples of actions directly related to creating or avoiding contact between objects and the body. For example, the blink reflex aims to avoid contact between a dangerous stimulus and the eye; thus, it is behaviourally useful that its magnitude depends on the likelihood that astimulus hits the eye, a likelihood that, in turn, depends on the proximity between the stimulus and the face (although by no means only on the proximity) 9, 54. Similarly, the stimulation of some multimodal VIP neurons elicits defensive postures aimed at preventing contact between a stimulus and a particular body part [77]. This variety of actions also explains why the PPS fields derived from, for example, the HBR and certain VIP neurons can be vastly different: HBR responses are increased when stimuli are near the face, whereas a specific VIP response might be stronger when the stimulus is near, say, the forearm [6]. It is clear why these measures are related to the physical space near the body (Box 1), but it is equally clear that these measures define a set of PPS fields of necessarily different shapes and sizes. In other words, the PPS field derived from a particular response will be shaped by whatever actions are linked to that response (Figures 2 and 4). Thus, the brain estimates the value of external events differently for the behaviour triggered by these events. This estimation is dependent on spatial location in egocentric coordinates, but the precise spatial relationship differs for the type of action required given a particular sensory event.

When considering certain PPS-related measures, the relation to actions aiming to create or avoid contact is less clear. For example, take the PPS field derived from reaction times to a tactile stimulus on the hand while an auditory stimulus approaches it. At first glance, the act of pressing a button might appear to have nothing to do with creating or avoiding contact between the hand and the source of the sound. Again, the interactive behaviour perspective helps resolve this issue, if one assumes that multiple simultaneous competing actions are being prepared at all times [71]. Thus, the entire set of potential actions related to contact between the hand and the sound source becomes more relevant with proximity to the hand. This interpretation is neurophysiologically plausible. Given that neural population coding of actions dictates that similar actions share network activity, any action triggered by stimulation of the hand (e.g., a button press) should share some of its network with those actions aimed at creating or avoiding contact between the hand and the sound source [78]. Therefore, as the sound approaches the hand, any hand-related actions are more readily enacted, resulting in shorter reaction times as a function of sound proximity. In this manner, most PPS fields likely display some summation of relevance of a set of actions, rather than a single action.

In fact, evidence from several PPS measures that do not immediately appear to be related to creating or avoiding contact also supports the perspective proposed here. One example comes from a reaction time study in which participants responded with a button press to stimuli delivered either to the hand, face, or torso. Expectedly, the proximity of looming auditory stimuli shortened reaction times for the tactile stimuli delivered to all these locations. By contrast, the proximity of receding auditory stimuli only shortened reaction times when tactile stimuli were delivered to the hand [38]. This clearly supports the framework provided here: many actions of the arm, face, and torso are relevant to avoiding a looming stimulus. However, there are more actions relevant to a receding stimulus executed by the arm (e.g., reaching to grasp) than by the torso or the head (except, perhaps, chest bumping or head butting). A second example is that, after tool use, cross-modal congruency is only strengthened near the tip of the tool, where contact with objects is made or avoided [79]. A final example comes from experiments investigating the effect of computer mouse use on reaction times to somatosensory stimuli delivered to the hand. Only during computer mouse use entailing making contact between the mouse cursor and some objects on the screen are these reaction times shortened when auditory stimuli are delivered near the computer screen where the mouse pointer is located [80]. All these examples indicate that the relevance of actions aiming to create or avoid contact determines PPS-related reaction times.

The observation that some PPS-related responses remain graded with proximity to a body part even when a transparent screen is placed between that body part and the stimulus (such as in [81]) could at a first glance suggest that such responses do not reflect contact-related actions. However, not all sensory information is considered at every stage of neural computation: neurons and networks underlying PPS measures are unlikely to have access to perfect situational information. In fact, many of these measures represent neural responses that are steps towards the ultimate selection of actions, and these steps, by definition, only take into account partial amounts of information, a principle neatly summarised in the affordance competition hypothesis [71]. Furthermore, defensive responses that rely mostly on early stages of processing offer a clear survival advantage: they do not need to integrate high-level information and, thus, can be more quickly and effectively enacted, at the cost of possibly having performed a useless action [81]. Intuitive examples of this point are common. While watching a movie or driving a car, sudden approaching objects on or behind the screen can often elicit overt avoidance responses, even though the person is (upon reflection, thus at the high cognitive level) fully aware of their safety.

The proposed reconceptualisation intuitively explains why factors other than proximity affect PPS. Consider how varying the context might affect the action choice of an animal. If the animal observes a rock rolling towards it with high speed, the animal would need to deploy a motor repertoire vastly different than if the object were moving tangentially to it. This would be the case even if the rock began in the same location (i.e., at the same distance from the animal). Environmental factors might also affect action choices and, thus, PPS fields. If an animal is faced with a predator, the animal might behave very differently if there were a large tree to climb, compared with if there were a small cave to scurry into. Thus, the PPS field derived from the same measure might be different depending on the contextual change represented by the presence of the tree or the cave (Figure 4). In fact, the proposed reconceptualisation also allows us to understand why and how PPS fields might interact within and between individuals [82]. For example, when considering a given individual surrounded by other agents, the relevance of the actions available to that individual depends not only on the position of other agents, but also on the set of actions available to them (i.e., their various contact-related PPS fields). Thus, social influences on PPS measures can be explained by postulating that we use our own coding of the contact-related action relevance of others as input to generate our own PPS fields.

Whether an action aims to create or avoid contact with a stimulus can also explain the fact that many PPS measures involve visuotactile and audiotactile integrations [83]. These contact-related actions link the perception of an object in external space, through sight or sound, to the perception of an object on the surface of the body, through touch. The idea of fields underlying such actions also resolves the apparent conflict with what, in the past, has been defined ‘appetitive’ versus ‘defensive’ PPS [84]. Indeed, both grasping and defensive actions have the common denominator of determining whether contact is created or avoided between an object and a body part, while occasionally being affected differently by variables such as object valence and movement.

Thus, the fact that factors other than proximity affect PPS fields arises naturally under our framework. This is in contrast to previous interpretations, which often considered these nonproximity effects as interesting exceptions to the rule. For example, when a biological measure considered to reflect PPS is altered by an experimental manipulation (e.g., a line bisection task is altered by tool use), it has often been concluded that ‘far becomes near’ 51, 55, or ‘peripersonal space expands’ 41, 47, 85. However, a modulation of a measure that depends, among other factors, on proximity says nothing about what nearness itself actually is, and concluding that ‘far becomes near’ or that ‘the PPS expands’ is, at least, a gross oversimplification. We argue that PPS fields are so dependent on nonproximity factors that proximity and nonproximity factors should no longer be considered separately. Rather, they should all be considered together as inputs to a set of functions that take into account many separate variables, one of which is proximity. This perspective of PPS as a set of continuous relevance-estimation fields by no means implies that the nervous system does not differentially process stimuli in near and far space, but does imply that there is no single strict boundary between the neural representations of events in ‘far’ and ‘near’ regions.

## Concluding Remarks

Many physiological measures have been linked to the concept of PPS because of their dependence on proximity to the body. However, not only is the concept of PPS ill-defined (Box 1), but the measures used to investigate PPS also: (i) do not define clear in-or-out zones; (ii) can be used to map out many different response fields; and (iii) are differently modulated by factors other than proximity. Here, we have provided a unifying perspective that accounts for all these facts: PPS can be understood as a set of continuous fields describing physiological responses, which reflect the behavioural relevance of actions aiming to create or avoid contact between objects and the body. This perspective fits into current systems-level understanding of brain function: parieto-premotor circuits describe the relevance of potential actions [71], and PPS fields reflect the relevance of a subset of these actions: namely, those dependent on proximity to the body due to their contact-related goal. Thus, this perspective on PPS also allows future research to integrate PPS findings with emerging new perspectives on hippocampal place and grid cells as state value predictors (see Outstanding Questions) [60]. Such future research will likely not only shed light on the interactions between egocentric and allocentric coding of space [61], but also allow a closer collaboration between neuroscience and robotics [86] by framing the issues faced by both fields in a common, practical language of action relevance in the real world.

Outstanding Questions What is the link between PPS fields and hippocampal place fields? A cursory glance suggests that they complement each other to deliver a full representation of space. However, there could be more to such a link, as suggested by combining recent advances in understanding the functional significance of place cells with the proposed perspective on PPS.

How necessary are PPS fields for interacting with the environment? Would it be possible to create an artificial agent able to move effectively without such response fields measurable from its internal machinery?

Can PPS fields be measured in species other than primates? Can one track the phylogenetic emergence of the hierarchical rules governing the relevance of contact-related actions across species?

Can PPS fields be derived during natural behaviour? What information would such ecologically more valid experiments provide?
